# Evaluation of Problematic Technology Use in Preschool Children

**DOI:** 10.1192/j.eurpsy.2024.421

**Published:** 2024-08-27

**Authors:** M. Tepetaş, A. Ünsal, G. S. Tepetaş Cengiz

**Affiliations:** ^1^Medical Faculty, Eskişehir Osmangazi University, Eskişehir; ^2^Mehmet Tanrıkulu Health Services Vocational School, Bolu Abant İzzet Baysal University, Bolu, Türkiye

## Abstract

**Introduction:**

Individuals have difficulty controlling their use of technology, it constantly on their minds when they don’t have access to it, it takes up too much time in their daily lives, and these situations negatively impact daily life are referred to as “problematic technology use”. The widespread use of technology from a young age is leading to an increase in the number of children with problematic technology use. Problematic technology use negatively impacts children’s development, especially their mental development. Important risk factors for problematic technology use include a long stay at home, a previous traumatic event, and low life satisfaction. In addition, it is possible that problematic technology use is more common in children with low social competence and low behavioral levels.

**Objectives:**

The aim is to determine the level of problematic technology use in 48-72-month-old children receiving preschool education, to examine some variables thought to be related to it, and to assess their level of social competence and behavior.

**Methods:**

The study was a cross-sectional research conducted between January and September 2023 among the parents of children studying in Eskişehir and Bolu. The study group consisted of the parents of 883 children. In our study, the Problematic Technology Use Scale for Young Children (PTUS-YC) and the Social Competence And Behavior Evaluation-30 Scale (SCBE-30) were used.

**Results:**

The age of the parents ranged from 20 to 54 years (mean: 35.5±4.8), 740 of them were women. The average age of the children was 63.2±7.3 months and 442 of them were boys. The scores obtained from PTUS-YC ranged from 26-104 and the mean was 55.1±14.9 points. Among the variables associated with problematic use of technology, those related to parents were place of residence, age and marital status, while those related to children were time spent at home with technological devices, parental control over content used, adaptation to school and ownership of a technological device. There is a weak positive correlation between children’s scores on the PTUS-YC and the SCBE-30 (r:0.336; p < 0.05).

**Conclusions:**

It can be said that problematic technology use in our study was at a moderate level. As the level of social competence and behavior increase, problematic technology use decreases. It is recommended to limit the time children spend with technological devices, ensure that parents control the content they use on technological devices, support their adaptation to school, and work on gaining social competence and positive behavior.

**Disclosure of Interest:**

None Declared

